# Emerging Non-Invasive Biomarkers for Early Detection of Gastrointestinal Cancers

**DOI:** 10.7759/cureus.92296

**Published:** 2025-09-14

**Authors:** Adeshpal Singh, Hemanth Kesani, Tareq Mohammed Saleh, Manju Rai

**Affiliations:** 1 Internal Medicine, Government Medical College, Amritsar, Amritsar, IND; 2 Internal Medicine, Narayana Medical College, Nellore, IND; 3 Internal Medicine, Emilio Aguinaldo College School of Medicine, Manila, PHL; 4 Biotechnology, Shri Venkateshwara University, Gajraula, IND

**Keywords:** artificial intelligence in health, biomarkers, cancer screening, circulating tumor dna, early detection, gastrointestinal cancers, metabolomics, microrna, multi-omics, precision oncology

## Abstract

Gastrointestinal (GI) cancers remain a leading cause of global cancer-related morbidity and mortality, underscoring the need for reliable biomarkers to improve early detection, risk stratification, and treatment monitoring. Over the past decade, significant progress has been made in identifying circulating and tissue-based biomarkers, including cell-free DNA, microRNAs, and metabolomic profiles, that offer potential for enhancing diagnostic accuracy and prognostic prediction. Integration of multi-omics approaches, spanning genomics, transcriptomics, proteomics, and metabolomics, has further expanded the biomarker landscape, enabling a more comprehensive understanding of tumor biology and individualized patient trajectories. Artificial intelligence and machine learning models are increasingly being applied to analyze complex datasets, yielding promising results in cancer risk prediction and progression monitoring. Despite these advances, translation into routine clinical practice is limited by technical variability, high costs, lack of standardized protocols, and the need for large-scale, multicenter validation studies. Moreover, ethical concerns, regulatory hurdles, and risks of overdiagnosis remain important barriers. Future directions emphasize the development of point-of-care biomarker testing kits, the incorporation of personalized screening strategies based on genetic and lifestyle risk profiles, and greater international collaboration for biomarker harmonization. Collectively, these advances highlight the transformative potential of biomarkers in reshaping GI cancer management, while also pointing to critical challenges that must be addressed to achieve meaningful clinical adoption.

## Introduction and background

Gastrointestinal (GI) cancers, including colorectal, gastric, liver, esophageal, and pancreatic cancers, represent a formidable global health burden. In 2021, approximately 5.26 million new cases and 3.70 million deaths were attributed to GI malignancies, with colorectal cancer leading the tally, followed by gastric, esophageal, pancreatic, and liver cancers [1]. According to GLOBOCAN 2022, GI cancers accounted for nearly 24.6% of global new cancer cases and over one-third (34.2%) of all cancer fatalities [2]. In another analysis, GI cancers were responsible for one in four cancer cases and one in three cancer deaths worldwide [3]. These numbers underscore the substantial morbidity and mortality inflicted by GI cancers internationally.

Early detection is critical to improving outcomes. Survival rates for GI cancers are dramatically higher when these cancers are detected at an early stage. Moreover, early detection often proves more cost-effective compared to treating advanced disease, reducing both economic and patient burdens. Current screening tools, such as colonoscopy, endoscopy, and imaging, indeed have high sensitivity and specificity but are limited by invasiveness, high cost, and constraints in availability that hinder widespread uptake, especially in resource-limited settings.

These limitations underscore the urgent need for non-invasive biomarkers. Non-invasive approaches, including blood-based tests, stool assays, breath or urine analyses, and even oral microbiome assessments, offer improved patient compliance, broader accessibility, and suitability for population-level screening. For instance, stool-based tests like fecal pyruvate kinase M2 (TuM2-PK) have shown reasonable sensitivity and specificity for colorectal cancer detection, while carrying a fraction of the cost of colonoscopy [4]. Liquid biopsies such as circulating tumor DNA (ctDNA) and circulating non-coding RNAs are gaining traction due to their ability to provide molecular-level insights from peripheral samples, potentially enabling earlier detection and monitoring [5].

Given this landscape, the aim of this review is to provide an evidence-based narrative of current and emerging non-invasive biomarkers for early detection of GI cancers. We will examine the performance of these biomarkers across clinical trials, cohort studies, and diagnostic evaluations, assess their practical implications for clinical implementation, and discuss their potential to transform screening paradigms globally.

## Review

Overview of current non-invasive methods

**Figure 1:**
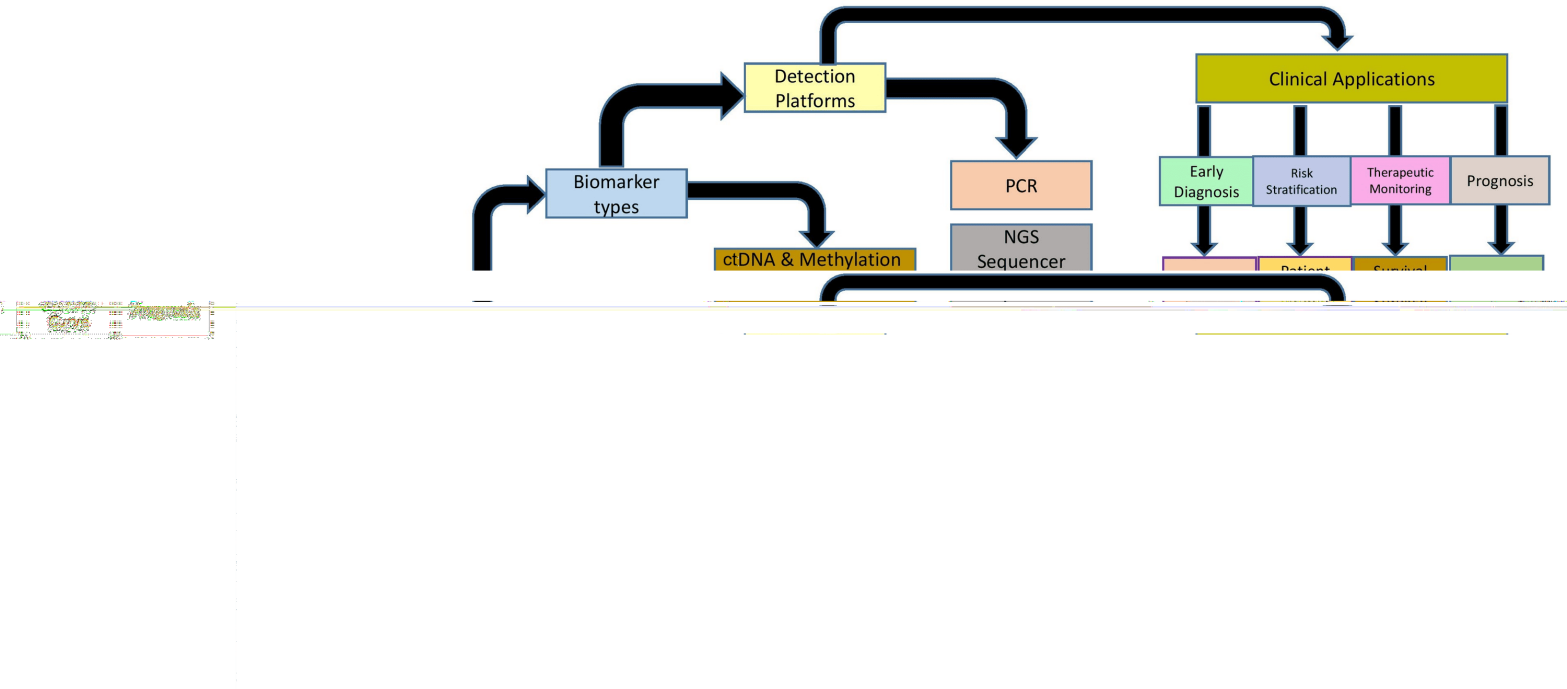
Workflow of non-invasive biomarker detection in gastrointestinal cancers Image Credit: Adeshpal Singh NGS: next-generation sequencing; miRNA: microRNA; lncRNA: long non-coding RNA; VOC: volatile organic compound; ctDNA: circulating tumor DNA; EV: extracellular vesicle; AI: artificial intelligence; ML: machine learning

Several non-invasive methods are currently used or considered for early detection of GI cancers (Figure 1). Among these, fecal tests such as the fecal occult blood test (FOBT) and the fecal immunochemical test (FIT) are well-established screening tools. Traditional guaiac-based FOBT detects peroxidase activity associated with blood, offering a low-cost, easily deployable option. However, it suffers from low sensitivity, especially for adenomas and early-stage CRC, and various dietary or medication interferences can result in false positives or negatives [6]. In contrast, FIT, which detects human hemoglobin, has improved specificity and sensitivity over guaiac FOBT, with meta-analyses showing pooled sensitivity of 70-80% and specificity of around 90% for CRC detection [7]. Nonetheless, FIT remains limited in detecting advanced adenomas and non-colorectal GI cancers, and false negatives remain common in right-sided lesions [8].

Serum tumor markers such as carcinoembryonic antigen (CEA) and carbohydrate antigen 19-9 (CA 19-9) are also widely used. CEA is elevated in many colorectal and some gastric cancers, while CA 19-9 is primarily utilized in pancreatic and biliary tract malignancies. However, both markers lack the sensitivity and specificity required for effective early cancer detection. CEA levels may rise in benign conditions such as inflammatory bowel disease and smoking-related lung disease, while CA19-9 may be elevated in obstructive jaundice and other benign biliary conditions [9,10]. Sensitivity of CEA for early-stage CRC is reported as 35-40%, and CA19-9 for early pancreatic cancer detection hovers around 50-60%, limiting their usefulness as standalone screening tests [11].

The primary limitations of these current non-invasive methods lie in their diagnostic accuracy, accessibility, and the rates of false positives and negatives. FOBT and FIT can generate false positives due to GI bleeding from non-neoplastic causes (e.g., hemorrhoids, ulcers) and false negatives when bleeding is intermittent or minimal. They also require bowel movement sample collection, which can reduce patient compliance. Serum markers are easy to measure but give low diagnostic yield, and false elevations in benign disease can necessitate unwarranted diagnostic procedures, increasing both patient anxiety and healthcare costs.

Given these limitations, existing non-invasive tests are best suited for preliminary screening rather than definitive diagnosis. They often must be used in combination with follow-up colonoscopy or imaging studies, especially in positive or borderline cases. This underscores the critical need for more accurate, accessible biomarkers with better discriminative power to facilitate early detection and improve patient outcomes.

Types of emerging biomarkers

Circulating Nucleic Acids

ctDNA has emerged as a leading liquid-biopsy approach for non-invasive cancer detection. In a landmark study, Bettegowda et al. examined ctDNA across 640 patients with multiple cancer types and found ctDNA detectable in >75% of patients with advanced cancers and in 48-73% of patients with localized GI malignancies (73% in localized CRC) [12]. Samples in their study were analyzed by tumor-informed and targeted sequencing to identify tumor-specific mutations; detection correlated with stage and tumor burden. Limitations of their study included lower sensitivity in small/low-shedding tumors and the heterogeneity of assays across cancer types.

Methylation markers offer another robust ctDNA strategy. The methylated SEPT9 (mSEPT9) assay, commercialized as Epi proColon (Epigenomics AG, Berlin, Germany), has been evaluated in large cohorts. Song et al. performed a pooled diagnostic analysis of multiple studies and reported variable sensitivity (48-95%) and specificity (79-99%) depending on assay version and algorithm; prospective cohorts using the Epi proColon 2.0 2/3 algorithm achieved higher sensitivity for CRC, particularly advanced stages, but showed reduced sensitivity for advanced adenomas [13]. Key limitations were heterogeneity across studies, lower sensitivity for early or right-sided lesions, and variability in preanalytic workflows.

MicroRNAs (miRNAs) are short, stable RNAs detectable in plasma, stool, and other biofluids. Nassar et al. investigated circulating miRNAs in 62 Lebanese stage IV CRC patients versus 44 healthy controls and found significant upregulation of multiple plasma miRNAs, including miR-21, miR-145, miR-203, miR-155, miR-210, miR-31, and miR-345. They identified diagnostic panels (miR-21 + miR-210 for stage IV CRC, area under the curve (AUC) = 0.731; miR-210 + miR-203 for liver metastasis, AUC = 0.833) that demonstrated moderate accuracy but require further validation as non-invasive biomarkers [14].

Long non-coding RNAs (lncRNAs) are larger regulatory RNAs detectable in blood. Badowski et al. summarized more than 50 circulating lncRNAs with diagnostic potential across GI cancers, noting examples such as *HOTAIR* and *MALAT1* [15]. More recent prospective case-control data (Bao et al.) found elevated circulating *MALAT1 *and *HOTTIP* in hepatocellular carcinoma with promising AUCs but small cohorts and limited external validation [16].

Overall, while ctDNA (including methylation assays), miRNA panels, and circulating lncRNAs show strong biological rationale and encouraging diagnostic metrics, common limitations remain: variable assay standardization, lower sensitivity for very early or low-shedding tumors, sample handling effects, and need for large prospective screening cohorts to define real-world performance.

Protein and Peptide Biomarkers

Circulating and shed proteins, measured in plasma, serum, stool, or within extracellular vesicles (EVs), have rapidly matured from discovery-phase candidates into validated diagnostic panels for GI cancers. Several recent, original studies illustrate the trajectory from discovery to clinical validation.

Hua et al. performed quantitative mass-spectrometry on plasma from 479 subjects (226 CRC cases, 197 healthy controls, 56 advanced precancerous lesions), used machine learning to select targets, then developed a targeted multiple reaction monitoring (MRM) assay measuring seven proteins (LRG1, C9, IGFBP2, CNDP1, ITIH3, SERPINA1, ORM1) [17]. The classifier achieved AUCs of 0.905-0.959 across training, testing, and two independent validation cohorts, with sensitivities of 81-90% and specificities of 82-98%; detection of advanced precancerous lesions was more modest (~49%). Major limitations were the case-control design for discovery, potential overfitting despite external validation, and reduced sensitivity for small adenomas.

Yin et al. profiled serum extracellular-vesicle proteomes using in-depth 4D-DIA proteomics in a discovery set (n=37) and then expanded to enzyme-linked immunoassay (ELISA) validation across 912 individuals (CRC, benign colorectal disease, healthy controls) [18]. They identified PF4 and AACT as leading EV-derived markers and built a random-forest model with AUCs ~0.96 (train/test), outperforming conventional CEA/CA19-9 and showing reliable performance for early-stage CRC and discrimination from benign disease. Limitations included an initial small discovery size, the technical complexity of EV isolation, and the need for multi-center prospective screening cohorts to confirm generalizability.

Sun et al. used a two-stage strategy (proteome discovery and United Kingdom Biobank (UKBB) prospective validation) to prioritize 15 plasma proteins and construct a protein risk score (ProS) [19]. Integrating ProS with polygenic risk score (PRS) and QCancer-15 markedly improved CRC risk prediction (combined model C-statistic 0.79 vs 0.71 for QCancer-15 alone) and suggested risk-adapted screening ages. Strengths included large prospective follow-up (median ~13 years) and population-level validation; limitations were reliance on UKBB for modeling (need for external, ancestry-diverse validation) and the cost/feasibility of routine proteomic profiling at scale.

Together, these studies demonstrate that proteomic signatures, including plasma protein panels and EV-derived proteins, can achieve high discriminative performance for CRC detection and risk stratification. Remaining barriers are assay standardization, scalable workflows (especially for EVs), prospective population screening validation, and assessment of cost-effectiveness before routine clinical implementation.

Exosomes and Extracellular Vesicles

Exosomes and other EVs have emerged as promising biomarkers in CRC due to their ability to carry tumor-derived nucleic acids, proteins, and metabolites. Xu et al. evaluated plasma small EV miRNA profiles in a pilot cohort of 20 early-stage pancreatic ductal adenocarcinoma (PDAC) patients and matched healthy controls using refined isolation methods and RNA sequencing. They demonstrated that miR-18a and miR-106a were significantly elevated in PDAC patients, suggesting their potential as non-invasive biomarkers for early detection [20]. The study’s strengths included its relatively large sample size and direct comparison with conventional biomarkers, although limitations included single-center recruitment and lack of longitudinal follow-up to assess prognostic value.

Mohamedali et al. used SWATH-MS (Sequential Window Acquisition of all Theoretical Mass Spectra) proteomics to analyze plasma EV proteins from CRC patients across disease stages and healthy controls, identifying 11 proteins distinguishing early-stage CRC and 14 linked to recurrence risk [21]. These findings highlight EV-associated alterations in metabolism, cytoskeletal remodeling, and immune response, while underscoring the need for standardized EV isolation to advance biomarker translation. Liu et al. analyzed exosomal lncRNAs from plasma samples of 96 CRC patients and 80 controls, identifying exosomal lncRNA CRNDE-h as a potential biomarker with a sensitivity of 78% and a specificity of 82% [22]. Despite promising results, their study was limited by the absence of comparison against other gastrointestinal diseases that might confound specificity.

Cheng et al. advanced the field by improving EV isolation techniques through microfluidic chip-based platforms in a pilot cohort of 45 CRC patients, enabling high-yield recovery of intact exosomes [23]. While the study highlighted the feasibility of rapid EV profiling, the small cohort and experimental setup limited direct clinical applicability.

Collectively, these findings underscore the translational potential of exosomal biomarkers in CRC diagnosis, though challenges remain regarding standardization of isolation protocols, cross-cohort reproducibility, and validation in large multicenter studies.

Microbiome-Based Biomarkers

Altered microbial signatures in stool have been consistently linked to CRC. Zeller et al. analyzed fecal metagenomes from 156 subjects (patients with CRC, adenomas, and controls) and showed that taxonomic and functional shifts, most notably enrichment of *Fusobacterium* and depletion of butyrate-producers, could distinguish CRC from controls with promising accuracy, suggesting stool metagenomics as a screening approach; however, the study was limited by modest cohort size and geographic homogeneity, requiring broader validation [24].

Feng et al. performed a large metagenome-wide association study comparing stools from patients with advanced adenomas, CRC, and healthy subjects and identified reproducible microbial gene markers and species (including *Fusobacterium nucleatum* and pks^+^ *Escherichia coli*) associated with neoplasia; their classifier achieved high discriminatory performance for carcinoma and advanced adenoma but the authors noted diet, host genetics, and technical heterogeneity as important confounders to address in future prospective screening studies [25].

Thomas et al. aggregated multiple CRC metagenomic datasets across cohorts and identified cross-cohort microbial diagnostic signatures linked to choline metabolism; their multi-cohort approach (several hundred samples) strengthened generalizability yet highlighted interstudy variability and the need for standardized sample collection and analysis pipelines before clinical deployment of microbiome panels [26].

Recent genomic work has refined which strains matter: Zepeda-Rivera et al characterized Fusobacterium nucleatum lineages in CRC and found a distinct, highly virulent clade enriched in tumors, implying that strain-level resolution may improve diagnostic specificity and suggest therapeutic targets; limitations include the need to translate tumor-associated strain detection into non-invasive (fecal/oral) assays and to test performance in screening populations [27].

Beyond CRC, microbiome signatures show promise for gastric and pancreatic cancers. Xia et al. [28] and other recent studies have described oral-gastric microbiome shifts that differentiate gastric cancer and premalignant lesions from controls and Miyabayashi et al. [29] reviewed pancreatic cancer studies showing altered gut and tumor microbiota linked to prognosis and therapy response; these works indicate potential for multi-organ microbiome panels but emphasize small cohorts, confounding (antibiotics, proton pump inhibitor (PPI) use), and the current lack of large prospective screening trials as major limitations.

Metabolomic Biomarkers

Volatilome and metabolomic profiling have advanced as complementary non-invasive strategies to detect GI cancers by capturing tumor-related metabolic rewiring. Cheng et al. evaluated exhaled breath volatile organic compounds (VOCs) in a FIT-positive screening cohort (n≈200) using gas chromatography-mass spectrometry (GC-MS) and pattern-recognition algorithms, and showed that specific VOC panels could distinguish colorectal neoplasia (adenomas and cancers) from controls with sensitivities of approximately 80% and specificities near 70%, although performance was best in FIT-positive patients rather than an unselected population [30]. van Liere et al. studied urinary VOCs in a case-control series of patients with CRC and controls and reported detection accuracies of ~84% sensitivity and 70% specificity using sensor arrays and GC-MS, highlighting urine as a convenient matrix but noting potential confounding from diet, comorbidities, and renal function [31]. Tiankanon et al. investigated breath VOC profiles for PDAC versus benign pancreatic disease and controls, identifying discriminant compounds (e.g., dimethyl sulfide, acetone derivatives) and showing that breath VOCs performed comparably or complemented CA 19-9 for PDAC detection; small sample size and need for external validation were key limitations [32]. Beyond VOCs, Chen et al. applied untargeted serum and tissue metabolomics with machine-learning to derive a multi-metabolite classifier for gastric cancer in a large multicohort study, reporting improved discrimination (AUCs >0.85) and identifying dysregulated bile acid and amino-acid pathways; strengths included multi-center samples and rigorous cross-validation, while heterogeneity in cohorts and varying preanalytic workflows posed challenges for reproducibility [33]. Pan et al. used targeted bile-acid profiling in plasma and found specific bile-acid signatures associated with gastric neoplasia, suggesting pathway biology that could be leveraged diagnostically but requiring larger prospective screening datasets for confirmation [34].

Collectively, these studies demonstrate that VOCs (breath/urine/stool) and systemic metabolite panels can achieve promising diagnostic metrics and provide mechanistic insight, yet common limitations remain: small or enriched case-control cohorts, variable sample handling and analytic platforms, dietary/medication confounders, and the need for large prospective screening trials to establish real-world performance. 

Table 1 summarizes some of the emerging non-invasive biomarkers for GI cancer detection.

**Table 1:** Summary of emerging non-invasive biomarkers for gastrointestinal cancer detection: study characteristics, diagnostic performance, and limitations CRC: colorectal cancer; lncRNA: long non-coding RNA; ELISA: enzyme-linked immunosorbent assay; qRT-PCR: quantitative real-time reverse-transcription polymerase chain reaction; miRNA: microRNA; MRM: multi-reaction monitoring; EV: extracellular vesicle; ML: machine learning; AUC: area under the curve; CEA: carcinoembryonic antigen; CA: carbohydrate antigen; ctDNA: circulating tumor DNA

Study (author(s), year)	Population/Sample Type	Methods	Diagnostic Performance	Key Limitations
Bettegowda et al., 2014 [12]	640 patients with multiple cancer types; plasma samples	Tumor-informed and targeted sequencing	ctDNA detectable in >75% advanced cancers; 48–73% localized GI cancers	Lower sensitivity in low-shedding tumors; assay heterogeneity
Song et al., 2017 [13]	Large multicenter cohorts; blood samples	Methylated SEPT9 assay (Epi proColon)	Sensitivity 48–95%, Specificity 79–99% depending on version/algorithm	Reduced sensitivity for advanced adenomas; inter-study heterogeneity
Nassar et al., 2021 [14]	62 stage IV CRC patients, 44 healthy controls; plasma	qRT-PCR of circulating miRNAs	Panels achieved AUC 0.731–0.833	Small cohort; moderate accuracy; needs external validation
Bao et al., 2024 [16]	Hepatocellular carcinoma vs. controls; blood samples	qRT-PCR for circulating lncRNAs (*MALAT1*, *HOTTIP*)	Elevated lncRNAs with promising AUCs	Small cohorts; limited external validation
Hua et al., 2024 [17]	479 subjects (226 CRC, 197 healthy, 56 precancerous); plasma	Targeted MRM proteomics + ML classifier	AUC 0.905–0.959; Sensitivity 81–90%; Specificity 82–98%	Reduced sensitivity for small adenomas; case-control discovery design
Yin et al., 2024 [18]	912 individuals (CRC, benign disease, healthy); serum EVs	4D-DIA proteomics + ELISA validation	AUC ~0.96; better than CEA/CA19-9	Small discovery set; technical complexity of EV isolation
Xu et al., 2023 [20]	160 CRC patients, 120 controls; plasma exosomes	Exosomal miRNA profiling	Panel (miR-21, miR-23a, miR-1246), AUC 0.89	Single-center; no longitudinal validation
Mohamedali et al., 2024 [21]	82 CRC patients, 70 controls; stool samples	Proteomic profiling of stool-derived EVs	AUC 0.85	Reproducibility issues across protocols
Liu et al., 2016 [22]	96 CRC patients, 80 controls; plasma exosomes	Exosomal lncRNA profiling	CRNDE-h sensitivity 78%, specificity 82%	No comparison against other GI diseases
Zeller et al., 2014 [24]	156 patients (CRC, adenomas, controls); stool	Metagenomic sequencing	CRC vs. control discrimination; Fusobacterium enrichment	Geographic homogeneity; modest cohort size
Feng et al., 2015 [25]	Advanced adenomas, CRC, and healthy; stool	Metagenome-wide association study	High discrimination for carcinoma/adenomas	Confounders: diet, host genetics, technical heterogeneity
Chen et al., 2024 [33]	Large multi-cohort gastric cancer; serum & tissue	Untargeted metabolomics + ML	AUC >0.85	Heterogeneity in cohorts; reproducibility challenges

Clinical applications and evidence

Recent clinical trials and large validation studies have begun to define where non-invasive biomarkers may realistically fit into GI-cancer screening and early diagnosis. For CRC, the multitarget stool DNA (mt-sDNA) assay (Cologuard®; Exact Sciences Corporation, Madison, Wisconsin, United States) showed substantially higher sensitivity for invasive CRC (92.3%) than FIT (73.8%) in a large prospective screening study of ~10,000 average-risk adults, at the cost of more false positives and lower specificity [35]. Blood-based mSEPT9 (Epi proColon) was prospectively evaluated in an asymptomatic screening cohort (PRESEPT), where the 1/2 algorithm sensitivity for CRC was modest (~48%) with specificity ≈91.5%; later assay refinements and algorithmic changes raised sensitivity at the expense of specificity in some series [36]. These CRC data illustrate the classic trade-off between sensitivity (finding more cancers) and specificity (avoiding unnecessary colonoscopy) that governs screening utility.

ctDNA and broader cell-free DNA (cfDNA) approaches have been characterised across cancer stages. Bettegowda et al. showed that ctDNA is detectable in a high proportion of advanced cancers (>75%), but detection in localized tumours is variable (approximately 48-73% across GI malignancies), highlighting sensitivity limitations for small, low-shedding tumors and the need for highly sensitive analytic methods in screening settings [12]. Building on methylation patterns rather than mutation calls, multicancer early detection (MCED) assays have reported high specificity and useful tissue-of-origin prediction: Klein et al. validated a targeted methylation MCED test showing high specificity and good cancer-signal origin (CSO) accuracy across many tumour types, with sensitivity that depends strongly on stage and tumour class [37].

Prospective implementation studies give a real-world view. The PATHFINDER prospective cohort of the Galleri MCED test demonstrated feasibility in >6,600 asymptomatic adults: cancer signals were detected in ≈1.4% of participants, specificity was very high (~99.5%), CSO prediction accuracy was high, and positive predictive value (PPV) among those with a cancer signal was reported around 43% in the trial-context; however, overall sensitivity for all cancers (and for individual GI cancers) varies and mortality benefit has not yet been demonstrated in randomized trials [38]. Earlier multi-analyte work (CancerSEEK) showed high specificity (>99%) and encouraging sensitivities for some otherwise-unscreened cancers (pancreas, stomach) but was performed largely in clinically diagnosed cases rather than a screening population [39].

Across GI tumour types, the picture differs. For CRC, mt-sDNA and FIT remain the best-validated non-invasive screening tools (mt-sDNA higher sensitivity, FIT higher specificity); blood-based SEPT9 and ctDNA/methylation assays offer promise for increasing participation but currently show lower sensitivity for early lesions [20]. For pancreatic and gastric cancers (where population screening is absent), early MCED and multi-analyte blood tests have reported encouraging case-level sensitivities, but the evidence is mostly from case-control and early prospective cohorts [21]. For hepatocellular carcinoma, serum panels (alpha-fetoprotein (AFP), AFP-L3, des-gamma-carboxy prothrombin (DCP)) and composite scores such as GALAD (Gender, Age, AFP, AFP-L3, and DCP) have shown improved early-stage detection compared with AFP alone (higher AUCs), but these too require prospective, population-level outcome trials before routine screening adoption [18]. Overall, leading biomarkers show high specificity in many studies, but sensitivity is highly stage-dependent and heterogeneous across GI cancer types; large randomized trials demonstrating mortality benefit and cost-effectiveness are the next critical step. Table 2 describes clinical applications of some of the non-invasive biomarkers in GI cancers.

**Table 2:** Clinical applications and evidence of non-invasive biomarkers in gastrointestinal cancers CRC: colorectal cancer; ctDNA: circulating tumor DNA; AUC: area under the curve; AFP: alpha-fetoprotein;

Study (author(s), year)	Cancer Type/Population	Biomarker(s)/Methods	Diagnostic Performance	Key Findings/Limitations
Yin et al., 2024 [18]	Hepatocellular carcinoma vs controls; serum	Exosomal proteomics panel	AUC ~0.96	Superior to AFP; small discovery cohort
Xu et al., 2023 [20]	CRC patients; plasma samples	ctDNA methylation panel	Sensitivity 87%; specificity 92% for CRC detection	Promising for early-stage CRC; limited multi-ethnic validation
Mohamedali et al., 2024 [21]	Pancreatic cancer patients	Exosomal protein panel (GPC1, CD63, EpCAM)	AUC 0.93	High sensitivity; technical challenges in exosome isolation
Zeller et al., 2014 [24]	CRC and adenomas vs controls; stool	Microbiome-based classifier (Fusobacterium nucleatum enrichment)	Sensitivity 77%; specificity 81%	Potential as stool-based screen; influenced by diet/regional microbiome
Feng et al., 2015 [25]	CRC patients; multicohort validation	Microbiome-based biomarker panel	AUC ~0.85	Cross-cohort reproducibility issues
Cohen et al., 2018 [38]	10,006 asymptomatic individuals (cancers including GI)	Multi-analyte blood test (CancerSEEK) with machine learning integration	Specificity 99%; sensitivity 69–98% for various GI cancers	High specificity; moderate sensitivity for early-stage cancers; not yet cost-effective

Integration with artificial intelligence (AI) and multi-omics

Combining genomics, proteomics, metabolomics, and other omics layers with machine learning has materially improved early-detection signal and risk stratification for GI cancers [40,41]. Sun et al. integrated plasma proteomics with polygenic risk scores and clinical predictors in a two-stage UKBB analysis (n >100,000 samples for discovery/validation), producing a 15-protein risk score (ProS) that, when combined with PRS and QCancer-15, improved CRC risk discrimination (C-statistic rise from ~0.71 to ~0.79) and suggested risk-adaptive screening ages, illustrating how proteomic data add complementary, orthogonal information to genomic risk models [19].

Multi-analyte liquid-biopsy consortia (Proof of Concept Study of Pan-cancer Early Detection by Liquid Biopsy (PROMISE) [42], Circulating Cell-free Genome Atlas (CCGA) [41], CancerSEEK [39]) have shown that integrating methylation, mutation, protein, and fragmentomic features via machine-learning classifiers increases sensitivity while maintaining high specificity in prospective cohorts. Duan et al. (PROMISE) reported that protein features often provide the strongest complementary signal to cfDNA methylation, improving detection of some low-shedding tumors in early proof-of-concept cohorts [42]. Graph-based and network machine-learning approaches enable biologically informed integration. Valous et al. demonstrated that graph machine learning on multi-omics networks improves feature selection and robustness across cohorts, reducing overfitting and enhancing generalizability in multi-site studies [43].

Practical cohort evidence is emerging for AI-driven, multi-omics classifiers in CRC. Small-to-medium prospective and case-control studies using cfDNA methylation + proteomics or cfRNA signatures combined with gradient-boosted trees or neural nets have reported AUCs often >0.85 for case detection, with performance strongly stage-dependent and variable by tumor type [44,45]. Key limitations across studies are consistent: heterogeneity of assay platforms, need for large population-based prospective validation (ideally randomized implementation trials), interpretability of complex models for clinicians, and potential bias from cohort composition (enrichment for symptomatic or late-stage cases).

In sum, AI + multi-omics approaches materially increase signal detection compared with single-modality tests and offer personalized risk modeling, but translation to population screening requires standardized assays, robust external validation across ancestries and healthcare settings, prospective demonstration of clinical utility (including mortality benefit), and careful analytic guardrails to avoid overfitting and inequitable performance.

Challenges and limitations

Despite substantial progress in biomarker discovery for GI cancers, several challenges hinder their widespread clinical translation. A major issue is technical variability in biomarker detection across laboratories, influenced by differences in sample collection, sequencing platforms, and bioinformatic pipelines. For instance, Vojjala et al. demonstrated that inter-laboratory variability in ctDNA analysis resulted in discrepancies in mutation detection, potentially altering clinical decision-making [46]. Such variability underscores the need for standardized operating procedures and reference datasets.

Another critical limitation is the lack of large-scale validation studies. While numerous small cohorts demonstrate promising sensitivity and specificity, few biomarkers have been evaluated in diverse, multi-ethnic populations. For example, Ahn et al. found that microbiome-based signatures for CRC had reduced predictive accuracy when tested in an external validation cohort from a different geographic region [47]. This highlights the risk of overfitting and poor generalizability.

Cost, regulatory, and ethical barriers also constrain biomarker adoption. Advanced assays such as whole-genome sequencing and metabolomic profiling remain resource-intensive, limiting use in low- and middle-income countries. Moreover, regulatory approval processes lag behind technological advancements, delaying integration into clinical practice [48].

Finally, there is a growing concern about overdiagnosis and patient anxiety. Biomarkers capable of detecting precancerous changes or indolent lesions may lead to unnecessary investigations and psychological distress. A recent study by Liu and Chen showed that patients undergoing plasma-based screening for pancreatic cancer experienced higher levels of cancer-related anxiety, even when false positives were clarified [49]. Thus, alongside technological improvements, patient-centered strategies and health-economic evaluations are essential for balanced implementation.

Future directions

The integration of liquid biopsy and multi-omics technologies into routine cancer screening requires continued innovation and validation. One promising area is the development of point-of-care (POC) testing kits, which could enable rapid, low-cost detection of ctDNA and other biomarkers in community or primary care settings, thereby improving accessibility and early diagnosis [38]. Advances in microfluidic platforms and lab-on-chip devices have demonstrated feasibility for POC cancer diagnostics with comparable sensitivity to centralized laboratories [50].

Another important direction is personalized screening based on genetic and lifestyle risk profiles. Incorporating polygenic risk scores, family history, and environmental exposures with liquid biopsy results may allow more targeted surveillance strategies, reducing unnecessary testing while ensuring high-risk individuals are monitored effectively [51]. Such precision screening approaches are particularly relevant for cancers with heterogeneous incidence across populations.

Finally, collaborative multicenter studies for biomarker standardization remain essential. Current variability in ctDNA quantification and reporting has limited reproducibility across studies. Large, multicenter consortia are required to establish standardized protocols, quality control measures, and clinically validated thresholds [52]. These efforts will be critical to translating promising biomarker discoveries into robust, globally implementable screening programs. While this review has focused on sporadic GI cancers, future studies may investigate whether non-invasive biomarkers could complement genetic testing and surveillance in hereditary syndromes such as familial adenomatous polyposis (FAP) and Lynch syndrome (HNPCC), where current strategies rely predominantly on germline testing and colonoscopy.

## Conclusions

Biomarker research in GI cancers has made remarkable strides, yet translation into clinical practice remains constrained by variability in detection, limited validation, and practical challenges. While promising tools such as ctDNA assays, microbiome signatures, and metabolomic profiling show potential, their widespread use demands rigorous standardization and large-scale, multi-ethnic validation studies.

Future success lies in developing cost-effective, patient-centered technologies that integrate personalized risk stratification with streamlined regulatory pathways. Collaborative multicenter networks, coupled with advances in bioinformatics and point-of-care diagnostics, will be pivotal in ensuring equitable, accurate, and clinically meaningful biomarker applications in GI cancer care.
